# *HER2* Alterations in Non-Small Cell Lung Cancer: Emerging Perspectives on the Therapeutic Landscape

**DOI:** 10.3390/ijms27052334

**Published:** 2026-03-02

**Authors:** Paola Muscolino, Elena Fassi, Diego Signorelli, Francesca Colonese, Diego Luigi Cortinovis, Giuseppe Lo Russo, Giulia Pasello, Carla Infurna, Giuliana Ciappina, Massimiliano Berretta, Vanesa Gregorc, Chiara Lazzari, Mariacarmela Santarpia

**Affiliations:** 1 School of Specialization in Medical Oncology, Department of Human Pathology “G. Barresi”, University of Messina, 98125 Messina, Italy; paola.muscolino@studenti.unime.it (P.M.); carla.infurna@studenti.unime.it (C.I.); 2 Department of Medical Oncology, Candiolo Cancer Institute—FPO—IRCCS, 10060 Candiolo, Italy; elena.fassi@ircc.it (E.F.); vanesa.gregorc@ircc.it (V.G.); chiara.lazzari@ircc.it (C.L.); 3 Medical Oncology Unit, Department of Medical and Surgical Specialties, Radiological Sciences, and Public Health, University of Brescia, ASST Spedali Civili, 25123 Brescia, Italy; 4 Niguarda Cancer Center, ASST Grande Ospedale Metropolitano Niguarda, 20162 Milan, Italy; diego.signorelli@ospedaleniguarda.it; 5 Medical Oncology Unit, Fondazione IRCCS San Gerardo dei Tintori, 20900 Monza, Italy; francescacolonese@live.it (F.C.); diegoluigi.cortinovis@irccs-sangerardo.it (D.L.C.); 6 Medicine and Surgery Department, University of Milano Bicocca, 20900 Milan, Italy; 7 Medical Oncology Department, Fondazione IRCCS Istituto Nazionale dei Tumori, 20133 Milan, Italy; giuseppe.lorusso@istitutotumori.mi.it; 8 Medical Oncology 2, Veneto Institute of Oncology IOV-IRCCS, 35100 Padova, Italy; giulia.pasello@iov.veneto.it; 9 Department of Surgery, Oncology and Gastroenterology, University of Padova, 35128 Padova, Italy; 10 Section of Experimental Medicine, Department of Medical Sciences, University of Ferrara, 44121 Ferrara, Italy; giuliana.ciappina@unife.it; 11 Applied Biology and Experimental Medicine, Department of Chemical, Biological, Pharmaceutical and Environmental Sciences, University of Messina, 98166 Messina, Italy; 12 Division of Medical Oncology, “Gaetano Martino” Hospital, 98125 Messina, Italy; massimiliano.berretta@unime.it; 13 Department of Clinical and Experimental Medicine, University of Messina, 98125 Messina, Italy; 14 Department of Human Pathology “G. Barresi”, University of Messina, 98125 Messina, Italy

**Keywords:** NSCLC, *HER2*, mAbs, TKIs, zongertinib, sevabertinib, ADCs, trastuzumab deruxtecan

## Abstract

Over the past decade, significant achievements in elucidating the molecular pathogenesis of non–small cell lung cancer (NSCLC) have catalyzed a paradigm shift from empiric therapies to precision oncology. In this context, *HER2* alterations, including protein overexpression, gene amplification, and activating mutations, constitute distinct molecular subsets within NSCLC. In the past few years, targeted therapeutic modalities such as antibody–drug conjugates (ADCs), particularly trastuzumab deruxtecan (the first agent to be granted FDA approval for *HER2*-mutant NSCLC), alongside selective tyrosine kinase inhibitors (TKIs), including zongertinib and sevabertinib, have demonstrated robust systemic efficacy and notable intracranial penetration. This comprehensive review delineates the molecular landscape and clinical phenotypes of *HER2*-altered NSCLC, synthesizes interim and mature data from ongoing clinical trials evaluating anti-*HER2* therapies, and critically examines efficacy and safety results from different classes of targeted agents. Further research is crucial to uncover potential mechanisms of resistance in NSCLC with *HER2* mutations and define sequencing or combinatorial strategies pertinent to optimizing individualized patient management.

## 1. Introduction

Lung cancer is the leading cause of cancer-related deaths, with the majority of cases attributable to cigarette smoking [1]. Non-small cell lung cancer (NSCLC) constitutes about 85% of lung cancer cases, and represents a highly heterogeneous disease, including multiple histological types, such as adenocarcinoma, squamous cell carcinoma, and large cell carcinoma. In recent decades, identifying several actionable genetic changes has revolutionized treatment approaches. The introduction of targeted therapies has shifted treatment from a “one size fits all” model to strategies based on specific molecular features, resulting in unprecedented advances in managing lung tumors [2,3]. Targeted drugs have significantly improved objective response rates (ORRs) and survival outcomes in patients with defined molecular profiles, usually with manageable side effects. The human epidermal growth factor receptor (HER) family consists of four related proteins: the epidermal growth factor receptor (EGFR, also known as HER1 or ErbB1), HER2 (ErbB2 or Neu), HER3 (ErbB3), and HER4 (ErbB4). Both *EGFR* and *HER2* are key drug targets, especially in NSCLC as well as breast and gastroesophageal cancers [4]. Each HER family receptor includes three main regions: an extracellular ligand-binding domain, a single α-helical transmembrane segment, and an intracellular tyrosine kinase domain.

*HER2* preferentially undergoes heterodimerization with other ErbB family members upon ligand binding to these receptors. The formation of *HER2*-containing dimers triggers receptor autophosphorylation and activates key downstream signaling cascades, predominantly the phosphatidylinositol-3-kinase (PI3K)/protein kinase B (AKT) and mitogen-activated protein kinase (MAPK) pathways, which play central roles in the regulation of cell proliferation, differentiation, and migration [4,5]. Different types of *HER2* alterations have been found in NSCLC, such as gene mutations (1–6.7%), gene amplifications (2–22%), and protein overexpression (7.7–23%). These alterations are often associated with poorer outcomes [6,7].

Therapeutic strategies targeting *HER2* in lung cancer have been under investigation for decades, with unsatisfactory results. Trastuzumab deruxtecan (T-DXd), approved in August 2022 by the Food and Drug Administration for patients with *HER2*-mutant NSCLC after prior systemic therapy, was the first agent to show significant benefit in this population [8]. Currently, platinum-based chemotherapy combined with immune checkpoint inhibitors (ICIs) remains the standard initial treatment for NSCLC patients with *HER2* mutations, providing an ORR of about 43% and median progression-free survival (PFS) of six months [9,10]. Despite this, new therapeutic options are emerging. Ongoing phase III trials in treatment-naïve *HER2*-mutant NSCLC may soon redefine current management strategies. This review discusses the latest developments and future directions in treating *HER2*-altered NSCLC.

A literature search was conducted using main medical research databases and on international cancer meetings websites: PubMed, Scopus, and Web of Science, and abstracts from the European Society for Medical Oncology (ESMO), American Society of Clinical Oncology (ASCO), and International Association for the Study of Lung Cancer (IASLC). The search included articles published between from 2004 to February 2026. Original research articles and relevant review articles published in English were considered eligible. The search strategy included combinations of the following keywords: (“HER2” OR “ERBB2”) AND (“non-small cell lung cancer” OR “NSCLC”) AND (“mutation” OR “amplification” OR “overexpression” OR “signaling pathways” OR “targeted therapy” OR “tyrosine kinase inhibitor” OR “antibody–drug conjugate”). The review specifically focused on prospective clinical trials and systematic reviews to synthesize the most relevant evidence.

## 2. HER2 Alterations in NSCLC

### 2.1. HER2 Mutations

**Figure 1:**
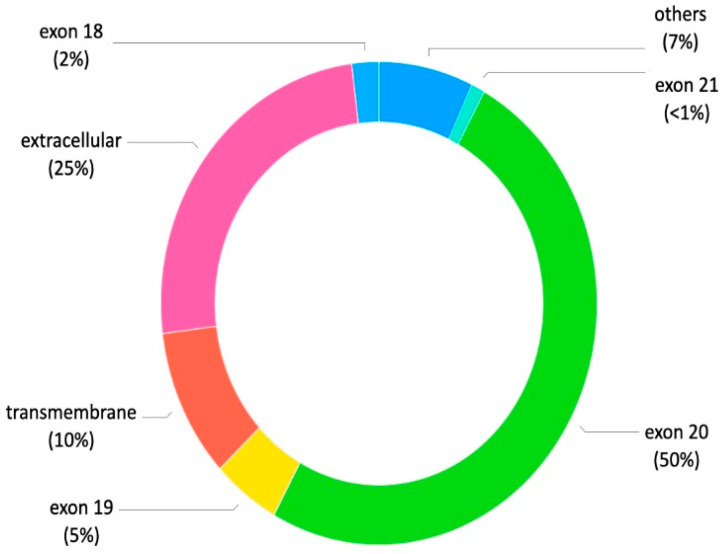
*HER2* mutations and frequency in patients with NSCLC.

The *HER2* gene, located on chromosome 17 (17q21), encodes a transmembrane growth factor receptor with intrinsic tyrosine kinase activity [5]. *HER2* mutations, found in about 4% of NSCLC cases, cause constitutive activation of its kinase domain (see Figure 1). These mutations can be identified trough different methodologies, including Sanger sequencing, next-generation sequencing (NGS), amplification refractory mutation system PCR (ARMS-PCR), or droplet digital PCR (ddPCR) [11]. However, NGS is recommended for *HER2* mutation testing due to its high sensitivity and ability to simultaneously analyze millions of DNA fragments while requiring limited input material. An optimal NGS platform should reliably detect the full spectrum of clinically relevant HER2 alterations, including exon 20 YVMA and non-YVMA insertions, missense mutations, copy number variations, and gene amplification, with low DNA input requirements, rapid turnaround time, and high reproducibility. Furthermore, *HER2* mutations can be detected on circulating tumor DNA (ctDNA) through plasma NGS. A recent analysis of the DESTINY-Lung01 and DESTINY-Lung02 studies demonstrated the analytical and clinical validity of the approved (United States and Japan) plasma-based Guardant360 companion diagnostic (CDx) *HER2*-mutated NSCLC for T-DXd treatment [12]. These mutations are more common in female patients, non-smokers, and with adenocarcinoma histology, similar to *EGFR* mutations. Generally, *HER2* mutations do not co-occur with other oncogenic drivers. The most frequent *HER2* mutation (about 34%) is a 12-base pair insertion in exon 20 (ex20ins) of the tyrosine kinase domain, adding YVMA between residues A775 and G776. Other recurrent exon 20 insertions affecting the kinase domain include G776delinsVC and G778_P780insGSP, accounting for 5.7% and 3.4%, respectively. Some mutations, like I655V (4.5%), P122L (2.3%), and G222C (1.1%), occur within the extracellular domain, while S310F (5.1%) affects the transmembrane domain [13]. The YVMA insertion variant, in particular, has been correlated with an increased incidence of brain metastases and poorer outcomes after chemotherapy compared to non-YVMA variants [5]. *HER2* mutations are key drivers in NSCLC and may contribute to acquired resistance during EGFR TKI therapy [5]. Figure 1 classifies the type and frequency of *HER2* mutations that may be observed in patients with NSCLC. Approximately 30% of patients develop brain metastatses [14]. A higher proportion of males was observed in the exon 20 group (41.9% vs. 75.0%, *p* = 0.025) compared with non-exon 20 mutations. Moreover patients carrying exon 20 mutations have a higher risk of lung and lymph gland metastasis. On the other hand, those with non-exon 20 mutations have a higher probability of developing metastasis to multiple organs [15].

### 2.2. HER2 Amplification

*HER2* amplification occurs in about 3% of NSCLCs. It is typically defined as a HER2/CEP ratio ≥ 2 or *HER2* gene copy number > 6, measured using fluorescence in situ hybridization (FISH) [13]. *HER2* copy number changes can be detected using various techniques, including NGS, real-time quantitative PCR (qRT-PCR), and FISH, with NGS being the most common in clinical practice due to its high resolution and the ability to analyze multiple genes at once and distinguish focal amplifications from broader chromosomal gains. qRT-PCR does not offer significant advantages over NGS, while FISH remains recommended in clinical studies [11].

Interpretation of FISH results in NSCLC generally follows breast cancer criteria: (i) *HER2*/CEP17 ratio ≥ 2.0, positive for *HER2* amplification; (ii) *HER2*/CEP17 ratio < 2.0, with *HER2* copy number ≥ 6.0 indicating positive amplification, <4.0 indicating negative, and 4.0–5.9 considered indeterminate; (iii) the presence of clustered *HER2* signals is considered unequivocally positive [11,16].

*HER2* amplification appears to occur more frequently in males and in patients with a smoking history, and it is associated with aggressive disease features, such as larger tumors, increased pleural spread and more lymphovascular invasion. Beyond its role as a primary oncogenic driver, *HER2* amplification also acts as an acquired resistance mechanism in *EGFR*-mutant NSCLC treated with *EGFR* TKIs, appearing in roughly 15% of patients who develop resistance [13].

### 2.3. HER2 Overexpression

HER2 overexpression has been identified in various solid tumors, including NSCLC. In lung cancer, studies have linked *HER2* overexpression to certain clinicopathological features such as male gender, adenocarcinoma histology (including papillary types), a history of smoking, and generally poorer prognosis [13,17].

The prevalence of HER2 overexpression ranges from 2.4% to 38%, commonly determined by immunohistochemistry (IHC) [18]. However, the relationships among *HER2* gene amplification, mutation, and protein overexpression in lung cancers remain controversial, indicating that these changes may constitute distinct clinical subtypes. The mechanism of overexpression is mainly associated with increased copy number from chromosome duplication and polysomy (*HER2* gene copy number higher than 5 or 6, but *HER2*/CEP17 < 2) [19]. HER2 protein levels are assessed through IHC using various scoring systems, such as those developed by the ASCO/College of American Pathologists (CAP) guidelines for breast cancer. These assign scores from 0 to 3+ based on membrane staining intensity. Scores of 0 and 1+ are considered *HER2*-negative, while 3+ denotes overexpression. A score of 2+ is considered equivocal in breast cancer and requires confirmation by in situ hybridization (ISH). In lung cancer, the proportion of IHC 2+ cases among FISH-positive tumors is relatively low, and an IHC score of 2+ shows limited sensitivity for identifying *HER2* amplification. Companion diagnostic criteria of *HER2* expression in NSCLC are based on IHC scoring. An IHC score of 0 is defined as *HER2*-negative. A score of 1+ is currently also classified as negative; however, in the context of the expanding clinical application of ADCs and emerging evidence, further validation is needed to clarify whether IHC 1+ should continue to be considered negative or instead be reclassified as *HER2*-low expression. In contrast, IHC scores of 2+ and 3+ are defined as *HER2*-positive [11].

## 3. *HER2* Status and Therapeutic Strategies

Over the past decade, the landscape of *HER2*-altered NSCLC management has changed considerably with the introduction of *HER2*-targeted therapies. Despite these therapeutic advances, the current standard first-line treatment for *HER2*-altered NSCLC is similar to the treatment for non-oncogene-addicted NSCLC and it usually consists of platinum-based chemotherapy combined with immune checkpoint inhibitors (ICIs) [20], although the impact of immunotherapy is controversial.

### 3.1. Immunotherapy

Immunotherapy represents a standard treatment option for patients with advanced NSCLC without actionable oncogenic drivers. However, their effectiveness is notably reduced in patients whose tumors harbor oncogenic alterations, such as *EGFR* mutations and *ALK* rearragements. This reduced efficacy is thought to result from a less inflamed tumor microenvironment and a generally lower tumor mutational burden (TMB) observed in this subgroup of patients. In particular, *HER2* mutations correlate with both low TMB and low PD-L1 expression, further limiting the response to ICIs in these patients [21]. Clinical studies evaluating the use of single-agent immunotherapy in *HER2*-mutant NSCLC have shown lower response rates, which range from 7.4% to 28.9%, according to various retrospective analyses [22,23,24,25,26]. Similarly, in unresectable stage III NSCLC following chemoradiation, consolidation durvalumab in *HER2*-mutants NSCLC was associated with less clinical benefit [27]. Furthermore, the combination of chemotherapy and immunotherapy in HER2-mutant NSCLC has yielded outcomes that are comparable to those seen in other NSCLC subgroups. Reported results include an ORR of 52%, a median PFS of six months, and an overall survival (OS) rate at one year of 88% [28]. Because of these findings, current guidelines from the European Society for Medical Oncology (ESMO) recommend the use of chemotherapy, with or without immunotherapy, as the first-line treatment for patients with *HER2*-mutant NSCLC [3].

### 3.2. HER2-Targeted Treatments

**Figure 2:**
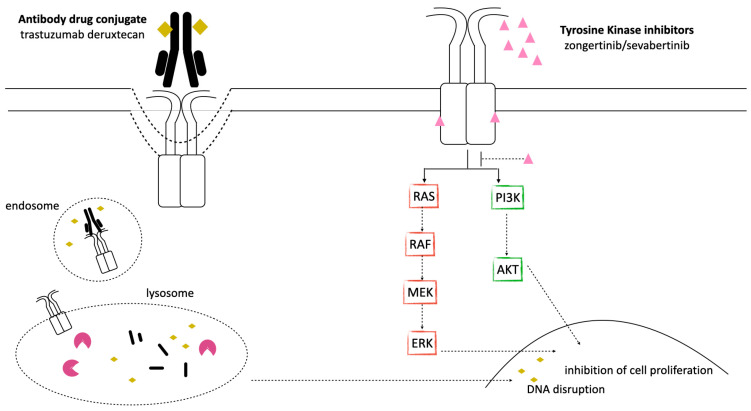
Mechanism of action of *HER2* inhibitors. After binding, trastuzumab deruxtecan undergoes internalization and intracellular cleavage, resulting in release of deruxtecan. Upon release, deruxtecan causes DNA damage and apoptotic cell death. Zongertinib and sevabertinib inhibit the kinase activity of *HER2* by engaging the ATP-binding pocket. As a consequence, the PI3K/AKT and MAPK signaling cascades are blocked, thus resulting in stopping the growth and survival of *HER2*-driven cancer cells.

Recent years have seen significant progress in the development and evaluation of various compounds targeting *HER2* alterations in NSCLC. These therapies include *HER2* Tyrosine Kinase Inhibitors (TKIs), monoclonal antibodies, and antibody–drug conjugates (ADCs). Each category encompasses different agents that have been investigated for their efficacy and safety in patients with *HER2*-altered NSCLC, as outlined in Table 1 and Figure 2.

#### 3.2.1. Monoclonal Antibodies (mAbs)

Trastuzumab, a humanized monoclonal antibody targeting the HER2 receptor, has been investigated in various chemotherapy combinations for the treatment of advanced, *HER2*-positive NSCLC. Despite these efforts, the outcomes have been largely disappointing. In a phase II trial assessing the combination of trastuzumab with carboplatin and paclitaxel in advanced NSCLC, selected according to *HER2* positivity (1+ to 3+ by Herceptest [Dako Corp, Carpinteria, CA], an ORR of 24.5% (95% confidence interval [CI], 13.8 to 38.3) was reported. The median PFS and median OS were 3.3 months and 10.1 months, respectively [32]. Additionally, the strategy of dual blockade using trastuzumab and pertuzumab was investigated in 15 patients carrying *HER2* mutations and 12 harboring *HER2* amplification who had previously received standard treatment. This approach resulted in an ORR of only 11% [33]. Given these limited benefits, *HER2* alterations in NSCLC were, for a long time, considered as an undruggable target, and further development of these treatment strategies was discontinued.

#### 3.2.2. Antibody–Drug Conjugates (ADCs) and Bispecific Antibodies (BsAbs)

Antibody–drug conjugates (ADCs) represent a novel class of antitumor agents designed to enhance the delivery of cytotoxic chemotherapy directly to cancer cells. These agents are composed of a monoclonal antibody, specifically targeting a tumor-associated antigen, chemically linked to a potent chemotherapy payload. The conjugation is achieved through a stable chemical linker, ensuring that the cytotoxic drug remains attached to the antibody until it reaches the targeted tumor cell. Upon binding to the tumor antigen, the ADC is internalized by the cancer cell. This facilitates the localized release of the cytotoxic agent, thus allowing a higher concentration of the chemotherapy drug to be delivered specifically to cancer cells, thereby minimizing exposure and potential toxicity to normal tissues. In addition to direct cytotoxicity, ADCs can stimulate immune cell effector functions and disrupt receptor dimerization, further contributing to their antitumor activity [34,35]. Among the ADCs developed for NSCLC harborig *HER2* alterations, trastuzumab emtansine and trastuzumab deruxtecan have demonstrated the first evidence of efficacy. Figure 2 summarizes the mechanism of action of ADCs.

#### 3.2.3. Trastuzumab Emtansine

Trastuzumab emtansine (T-DM1) is an ADC that combines the *HER2*-targeted monoclonal antibody trastuzumab with the cytotoxic microtubule inhibitor emtansine (DM1). The clinical efficacy of T-DM1 has been evaluated in a phase II study including pretreated populations selected for *HER2* overexpression. In one study, patients were included if they exhibited *HER2* expression by IHC 2+ with FISH positivity, or IHC 3+ status. The results demonstrated limited effectiveness, with an ORR of only 7% (1 out of 15 patients). Notably, the only patient who responded carried a *HER2* mutation within exon 20 [36]. The efficacy of T-DM1 was explored in another phase II clinical trial, which enrolled 49 patients with advanced HER2-overexpressing NSCLC, previously treated with platinum-based chemotherapy. Of these patients, 29 had IHC 2+ tumors and 20 had IHC 3+ tumors. No objective responses were observed in the IHC 2+ cohort. In contrast, the IHC 3+ cohort achieved an ORR of 20%, with responses particularly noted among patients harboring *HER2* gene amplification [37].

A phase II basket trial including *HER2*-mutant NSCLC showed more promising results, with partial responses observed in 8 out of 18 patients, median duration of response (DoR) of 4 months (range, 2 to 9 months), and a median PFS of 5 months (95% CI, 3 to 9 months). Treatment-related adverse events were generally grade 1 and 2. The most common were transaminase elevation (44%), thrombocytopenia, fatigue, and nausea (33%) [38]. Updated data from 28 pretreated patients reported an ORR of 50%. In *HER2*-amplified NSCLC, the ORR observed was 55% [39].

#### 3.2.4. Trastuzumab Deruxtecan

Trastuzumab deruxtecan (T-DXd) is an ADC consisting of the humanized monoclonal antibody trastuzumab covalently linked to the topoisomerase I inhibitor deruxtecan through a tetrapeptide cleavable linker. Initially developed in patients with *HER2*-amplified breast cancer, it is the first approved *HER2*-directed therapy for patients with previously treated, advanced, *HER2*-mutated NSCLC. Encouraging results were reported in a phase I trial enrolling 11 pretreated patients carrying *HER2* mutations, with an ORR of 72.7% (95% CI 39–94), a median PFS of 11.3 months (95% CI 5.5–14.3), and median DoR of 9.9 months (95% CI 6.9–11.5) [40].

During the dose escalation phase, no dose-limiting toxicities (DLTs) were observed, and the dose-expansion phase evaluated T-DXd in patients with *HER2*-positive breast or gastric cancer at the dose of 5.4 or 6.4 mg/kg intravenously once every 3 weeks. In the other cohorts, the maximum tolerated dose (MTD) was defined at 6.4 mg/kg and was therefore selected for the subsequent drug development [41].

DESTINY-Lung01 was an open-label, two-cohort, phase II study, designed to evaluate the efficacy and safety of T-DXd in previously treated NSCLC patients carrying *HER2* mutations. T-DXd was administered at the dose of 6.4 mg/kg. Ninety-one *HER2*-mutant NSCLC patients were enrolled. Results showed an ORR of 55% (95% CI 44–65), a median PFS of 8.2 months (95% 6.0–11.9), and a median OS of 17.8 months (95% 13.8–22.1). However, 41% of patients reported grade ≥ 3 treatment-related adverse events (TRAEs), including hematologic and gastrointestinal toxicities. Interstitial lung disease (ILD) occurred in approximately 26% of patients. Of these, four cases of grade ≥ 3 ILD were observed, two of which were fatal [42].

In order to reduce the risk of ILD, the phase II DESTINY-Lung02 trial was designed to compare the safety and efficacy of T-DXd when administered at the dose of 6.4 mg/kg over 5.4 mg/kg in 152 previously treated *HER2*-mutant NSCLC. Results demonstrated that the dose of 5.4 mg/kg determined an ORR of 49.0% (95% 39–59.1), a median PFS of 9.9 months (95% 7.4-NE), and a median OS of 19.5 months. The incidence of T-DXd-induced ILD/pneumonitis was significantly lower at the dose of 5.4 mg/kg (12.9%) compared to a 6.4 mg/kg dose (28.0%) [8]. The updated analysis of the study confirmed the T-DXd activity in patients receiving 5.4 mg/kg dosage, with a median PFS of 10.0 months (CI 95%, 7.7 to 15.2) and a median OS 19.0 months (CI 95%, 14.7-NE) [40]. Drug-related ILD occurred at a higher frequency in the higher dose arm (14.9% vs. 32.0% in the 5.4 mg/mg and 6.4 mg/kg arms, respectively), although most of these events were grade 1 or 2, with one grade 5 in each arm. Of note, health-related quality of life was preserved for the duration of T-DXd treatment [43].

In a post hoc secondary pooled analysis of the DESTINY-Lung01 and DESTINY-Lung02 trials, Jänne et al. evaluated the efficacy and safety of T-DXd according to the dose (5.4 mg/kg vs. 6.4 mg/kg) in patients with previously treated *HER2*-mutant NSCLC with or without untreated or previously treated stable brain metastases (BMs) (Table 1). Patients with and without BMs who received T-DXd at the dose of 5.4 mg/kg (102 pts) had confirmed ORR of 47% (95% CI, 29–65%) and 50% (95% CI, 38–62%), respectively, and an intracranial confirmed ORR of 50% (95% CI, 23–77%). An ORR of 50% (95% CI, 36–64%) and 59% (95% CI, 48–69%) was observed in patients receiving T-DXd at the dose of 6.4 mg/kg with and without BMs, respectively, with an intracranial confirmed ORR of 30% (95% CI, 15–49%). These data suggest that T-DXd, at the approved dose of 5.4 mg/kg, is an effective treatment option for patients previously treated, with or without BMs [44]. Based on comparable efficacy and a reduced risk of ILD, the 5.4 mg/kg dosage was selected for the ongoing phase III DESTINY-Lung 04 study (NCT 05048796), designed in treatment-naïve, *HER2*-mutant patients. Four hundred fifty nine metastatic patients have been randomized between T-DXd and cisplatin/carboplatin, pemetrexed, and pembrolizumab. Primary endpoint is PFS. Results from this trial may establish T-DXd as the new first-line therapeutic standard for this molecular subset of NSCLC [45]. To improve its antitumor efficacy, several studies are evaluating the combination of T-DXd with ICIs or platinum-based chemotherapy.

Planchard et al., in a phase Ib trial, DESTINY-Lung03, evaluated T-DXd as monotherapy or in combination with durvalumab and platinum-based chemotherapy in patients with pretreated, metastatic *HER2*-overexpressing NSCLC. Part 1 evaluated T-DXd 4.4 or 5.4 mg/kg plus durvalumab (1120 mg) and cisplatin (60 or 75 mg/m^2^; Arm 1A)/carboplatin (AUC 4 or 5; Arm 1B); or T-DXd 5.4 mg/kg monotherapy (Arm 1D). The primary endpoints included DLTs and AEs (Arms 1A and 1B), while safety (Arm 1D) and efficacy (all arms) were secondary endpoints. On April 1, 2024, at the data cutoff, 11, 24, and 36 patients had received treatment in the cisplatin-based combination arm, the carboplatin-based combination arm, and the T-DXd monotherapy arm, respectively. Combination regimens were associated with a high incidence of DLTs, primarily hematologic, such as febrile neutropenia reported in Arm 1A: (*n* = 1; Grade [G]5; 4.4 mg/kg/1120 mg/60 mg/m^2^ doses) versus febrile neutropenia observed in Arm 1B: *(n* = 1; G3; 4.4 mg/kg/1120 mg/AUC 5 doses; *n* = 1; G4; 4.4 mg/kg/1120 mg/AUC 4 doses) with the occasional occurrence of severe and fatal events. Drug-related serious AEs occurred in 63.6%, 37.5%, and 16.7% of Arms 1A, 1B, and 1D, respectively. Confirmed ORR were 37.5% (95% CI: 18.8–59.4) in Arm 1B and 44.4% (95% CI: 27.9–61.9) in Arm 1D, demonstrating the substantial clinical activity of single-agent T-DXd, and suggesting that the combination regimens are not recommended for this population [46]. Novel agents are currently under investigation, including trastuzumab rezetecan or zanidatamab.

#### 3.2.5. Trastuzumab Rezetecan (SHR-A1811)

Trastuzumab rezetecan is an ADC consisting of the humanised *HER2*-trastuzumab, linked to the DNA topoisomerase I inhibitor SHR169265 via a tetrapeptide-based cleavable linker. The phase II HORIZON-Lung trial, conducted in China in 94 patients with previously treated locally advanced or metastatic NSCLC carrying an activating *HER2* mutation, demonstrated an ORR of 73% (95% CI, 63.3–82.0) and a median PFS of 11.5 months (95% CI 9–9 not reached). Data on OS are not yet mature. Treatment was effective also in patients with brain metastases (ORR: 87.5%) [47].

#### 3.2.6. Zanidatamab (ZW25)

Zanidatamab (ZW25) is a humanized bispecific monoclonal antibody (bsAb) targeting two *HER2* extracellular domains: the dimerization domain (ECD2) and the juxtamembranous one (ECD4). In a phase 1 dose escalation and expansion study, including patients with locally advanced or metastatic *HER2*-expressing or *HER2*-amplified solid tumours, ZW25 showed a good safety profile. The most common adverse events were diarrhea (43%) and infusion-related reactions (34%), and only 3% of toxicities were grade 3/4. In Part 2, enrolling 86 patients with *HER2* overexpression or amplification, the ORR was 37%, with a DOR of 8.5 months in the biliary tract cancer group, 5.6 months in the colorectal cancer group, and 9.7 months in the group of other cancers. The median PFS for all patients was 5.4 months (95% CI 3.7–7.3) [48].

Novel ADCs are under evaluation in phase I/II clinical trials for NSCLC, including BL-M17D1 (NCT06114511), TQB2102 (NCT06496490), and MRG002 (NCT05141786). Additional ADCs are being assessed in phase I trials across multiple solid tumors, including NSCLC, such as DB-1303/BNT323 (NCT05150691), XMT-2056 (NCT05514717), and GQ1001 (NCT04450732) [49].

#### 3.2.7. Tyrosine Kinase Inhibitors

Several pan-HER TKIs have been extensively tested in *HER2*-altered NSCLC patients, including afatinib, neratinib, dacomitinib, and poziotinib, with disappointing results, probably due to DLTs or suboptimal potency [23,50,51,52,53].

In contrast with pan-HER inhibitors, novel mutant-selective TKIs have been developed with the aim of improving activity against *HER2* mutations, while reducing off-target side effects. Zongertinib and sevabertinib represent the first *HER2*-selective TKIs that have demonstrated significant clinical activity in this setting (Table 1). Figure 2 summarizes the mechanism of action of *HER2* TKIs.

#### 3.2.8. Zongertinib

Zongertinib is an oral, irreversible TKI that selectively inhibits *HER2* while sparing wild-type receptors. Preclinical data suggest its activity against *HER2*-dependent human cancer cells resistant to trastuzumab deruxtecan and in *KRAS* G12C mutant NSCLC cell lines resistant to KRAS inhibitors [54]. This agent was evaluated in the Beamion LUNG-1 study, a multicohort, phase Ia/Ib trial enrolling 200 naïve or previously treated patients with metastatic NSCLC carrying TKD and non-TKD *HER2* mutations. The primary endpoint was ORR, assessed by blinded independent central review (cohorts 1 and 5) or by investigator review (cohort 3). Secondary endpoints included DoR and PFS. Zongertinib achieved a confirmed ORR of 71% (CI 95% 60–80) in 75 previously treated patients carrying TKD mutations (cohort 1), with a median DoR of 14.1 months (CI 95% 6.9—not estimable) and a median PFS of 12.4 months (CI 95% 8.2—not estimable). The ORR in 31 patients previously treated with *HER2*-directed ADCs (cohort 5) was 48% (CI 95% 32–65), while that in those with non-TKD mutations (cohort 3) was 30%. Data from thr 74 naïve patients (cohort 4) demonstrated an ORR of 77% (CI 95% 66- 85), with a median DoR of 9.7 months (CI 95% 7.1–9.9) and a median PFS of 11 months (CI 95% 9.7–12.4) [55]. Twenty seven patients had brain lesions. An intracranial response of 41% (95% CI 25–59) and an intracranial disease control rate of 81% (95% CI 63–92) were reported, thus demonstrating the notable intracranial activity of zongertinib. Treatment was generally well tolerated. Grade ≥ 3 adverse events were reported in 17%, 3%, and 25% of patients in cohorts 1, 5, and 3, respectively. No cases of drug-related interstitial lung disease were observed [56].

On August 8, 2025, the Food and Drug Administration granted accelerated approval to zongertinib for adults with unresectable or metastatic NSCLC whose tumors harbor *HER2* TKD-activating mutations, as detected by an FDA-approved test, and who have received prior systemic therapy [30]. The phase III ongoing Beamion LUNG 2 study (NCT06151574) is currently evaluating the efficacy and safety of zongertinib in treatment-naïve NSCLC carrying TKD *HER2* mutations. Two hundred seventy patients, stratified according to the type of mutations, are randomized between zongertinib or platinum pemetrexed pembrolizumab combinations. The primary end point is PFS. Zongertinib is also under evaluation in the adjuvant setting in the phase 3 trial Beamion-LUNG 3 (NCT07195695).

#### 3.2.9. Sevabertinib

Sevabertinib is an oral, reversible TKI that potently inhibits tumors with *EGFR* and *HER2* mutations, including ex20ins, while sparing wild-type *EGFR*. Preclinical data demonstrate that activities of sevabertinib and zongertinib are comparable in cancer cell lines carrying *HER2* alterations, while zongertinib has no activity against exon 20 *EGFR* insertions [57]. Clinical efficacy and safety of sevabertinib was tested in the phase I-II SOHO-01 trial in 209 treatment-naïve and previously treated NSCLC patients carrying TKD and non-TKD mutations. Patients were stratified into three cohorts based on prior therapy. Cohort D included those who had received prior treatment, but not *HER2*-targeted therapy. Cohort E included those who had received *HER2*-directed ADCs. Cohort F included treatment-naïve patients. The median follow-up was 13.8 months in cohort D, 11.7 months in cohort E, and 9.9 months in cohort F. In cohort D (*n* = 81), an ORR of 64% (CI 95% 53–75), a median DOR of 9.2 months (CI 95% 6.3–13.5), and a PFS of 8.3 months (CI 95% 6.9–12.3) were reported. In cohort E (*n* = 55), sevabertinib demonstrated an ORR of 38% (CI 95% 25–52), with a median DOR of 8.5 months (CI 95% 5.6–16.4) and median PFS of 5.5 months (CI 95% 4.3–8.3). In cohort F (*n* = 73), ORR was 71% (CI 95% 59–81), median DOR was 11.0 months (CI 95% 8.1-not estimable), and median PFS was not reached. In 22%, 27%, and 12% of patients enrolled in cohorts D, E, and F, respectively, BMs were present. In cohort D, ORRs were 61% in patients with brain metastases and 65% in those without. In cohort E, ORRs were 27% in patients with BMs and 43% among those without, whereas in cohort F, ORRs reached 78% and 70% in patients with and without BMs, respectively. In patients with BMs at baseline, post-baseline intracranial progression was observed in 22% of cohort D, 20% of cohort E, and 0% of cohort F. In contrast, in patients without BMs at baseline, post-baseline brain progression occurred in 6% of cohort D, 5% of cohort E, and 3% of cohort F. Diarrhea was the most common adverse event (reported in 84–91% of patients), with grade ≥ 3 events observed in 5–23% of patients [58]. An exploratory analysis from the SOHO-1 trial revealed activity across *HER2* variant classes. Higher response rates were observed in tumors harboring TKD mutations, particularly the Y772_A775dup (YVMA) mutation versus patients with *HER2* non-TKD mutations. On November 19 2025, the FDA granted accelerated approval to sevabertinib [31]. The Oncomine Dx Target Test (Life Technologies Corporation) as a companion diagnostic test to detect *HER2* TKD activating mutations in patients with non-squamous NSCLC was also approved.

The ongoing phase III SOHO-02 study (NCT06452277) is evaluating the efficacy of sevabertinib in 278 patients with naïve advanced NSCLC harboring TKD *HER2* mutations. Patients are randomized between sevabertinib over platinum pemetrexed pembrolizumab and the primary end point is PFS.

## 4. Mechanisms of Acquired Resistance to *HER2* Inhibitors

Little is known about the acquired mechanisms of resistance to *HER2* inhibitors. Data from *HER2*-mutant tumor cell lines, patient-derived xenografts, and tumor tissues were collected to identify acquired resistance mechanisms to T-DXd [59]. Alterations of the deruxtecan payload, mediated by loss of Schlafen Family Member 11 (SLFN11), a DNA/RNA helicase, or copy number gain in ABCC1, responsible for the transcription of the efflux pump, MRP1, or the blockade of MRP1, have been described as some of the acquired mechanisms to T-DXd. Furthermore, secondary *HER2* mutations determining a steric hindrance to trastuzumab binding were observed. Data from liquid biopsy performed in *HER2* mutant NSCLC patients progressing on T-DXd showed the activation of alternative signaling pathways, including *MET*, *FGFR2* mutation, and *EGFR* and *BRAF* amplifications. Preclinical data indicate that these mechanisms do not affect the sensitivity to *HER2* TKIs, as zongertinib, thus suggesting that this class of drugs might be effective to overcome acquired resistance to T-DXd.

## 5. Perspectives and Conclusions

*HER2* mutations, amplifications, and overexpression display distinct biological characteristics and variable responses to treatments, highlighting the importance of an accurate molecular diagnostics. Platinum-based chemotherapy and immunotherapy remain standard first-line options. However, the emergence of novel *HER2*-targeted therapies, including zongertinib and sevabertinib, and potent ADCs such as trastuzumab deruxtecan has led to meaningful durable responses and survival benefits in patients with *HER2*-mutant NSCLC, thus changing the treatment paradigm for this subset of oncogene-driven tumors. The recent approval of anti-*HER2* targeted agents supports the use of a comprehensive molecular profiling with NGS techniques in routine clinical practice to identify these mutations and tailor treatment strategies at the time of tumor diagnosis. In addition, HER2 overexpression identifies a subset of patients that might benefit from T-DXd-based therapies, and currently ongoing studies will provide definitive conclusions on the efficacy of this strategy.

Increasing evidence suggests the clinically meaningful intracranial activity of anti-*HER2* agents. However, the optimal treatment algorithm still remains to be defined. How to combine these agents with loco-regional treatments needs to be further explored. The efficacy observed in the phase I/II clinical trials, exploring the anti-tumor activity of T-DXd, zongertinib, and sevabertinib in patients with *HER2*-mutant NSCLC has opened new questions regarding the therapeutic strategy to use (first T-XDd followed by *HER2* TKIs or *HER2* TKIs followed by T-XDd). Considering the different safety profile of these agents and the different mechanisms of action, a sequential use might be possible and currently ongoing studies will provide additional evidence.

Multiple biological mechanisms of acquired resistance to T-XDd have been identified. Based on preclinical evidences, *HER2*-mutated cancer cells progressing to T-XDd might retain sensitivity to *HER2* TKIs. Data from phase I/II clinical trials suggest the efficacy of zongertinib and sevabertinib following T-XDd, but further research is warranted in order to achieve maximal clinical benefit and improve patients’ outcomes.

With the advent of new targeted therapies specifically targeting *HER2* mutations, the prognosis for patients with *HER2*-mutant NSCLC, historically considered dismal, is remarkably improving. However, active areas of research include the discovery of genetic alterations underlying resistance and the optimal management of anti-*HER2* toxicities, more pronounced for the ADCs compared to the TKIs, in the clinical setting.

## Figures and Tables

**Table 1:** *HER2* inhibitors, already approved by the Food and Drug Administration (FDA) in previously treated *HER2*-mutated NSCLC patients. ADC: antibody–drug conjugate; TKI: tyrosine kinase inhibitor; RD: recommended dose; NE: not estimable; NA: not available.

	Trastuzumab Deruxtecan	Zongertinib	Sevabertinib
Mechanism of action	ADC: a humanized anti-*HER2* monoclonal antibody linked to a topoisomerase I inhibitor payload through a tetrapeptide-based cleavable linker	Oral irreversible TKI that selectively inhibits *HER2*	Oral reversible TKI with activity against *HER2* insertions and missense mutations
Clinical trial	DESTINY-Lung02 (Phase 2)	Beamion LUNG-1 (Phase 1a/1b)	SOHO-01 study (Phase 1/2)
Population	102 NSCLC pts refractory to standard treatment, with *HER2* TKD and non-TKD mutation	126 NSCLC pts previously treated, *HER2*-mutant (TKD mutation: cohort 1 and 5; non-TKD mutation: cohort 3) and 74 treatment-naïve, *HER2*-mutant (TKD mutation: cohort 2)	209 NSCLC pts naïve or previously treated, with *HER2* (TKD and non-TKD mutations
Brain metastases	35/102 (34.3%)	- Pre-treated (cohort 1): 28/75 (37%) - Naïve: 22/74 (30%)	42/209 (~20%; all cohorts)
FDA approval	August 2022 [29]	August 2025 [30]	November 2025 [31]
G ≥ 3 toxicity (%) at the RD	neutropenia (18.8%) anemia (10.9%)	ALT/AST increased (13%)	diarrhea (23%)
ORR (95% CI)	49% (39–59.1)	- Pre-treated (cohort 1): 71% (60–80); - Pre-treated (cohort 3): 30%; - Naïve (cohort 2): 77% (66–85); - Prior HER2-ADC (cohort 5): 48% (32–65)	- Pre-treated: 64% (53–75); - Naïve: 71% (59–81); Prior *HER2*-ADC: 38% (25–52)
PFS months (95% CI)	10.0 (7.7–15.2)	- Pre-treated (cohort 1): 12.4 (8.2-NE); - Pre-treated (cohort 3): NA; - Naïve (cohort 2): 11 (9.7–12.4); - Prior HER2 ADC (cohort 5): 6.8 (NA)	- Pre-treated: 8.3 (6.9–13.5); - Naïve: 11 (8.1-NE); - Prior *HER2* ADC: 5.5 (4.8–8.3)
Ongoing phase III trials in naïve pts	Destiny Lung 04	Beamion Lung 2	SOHO-02

## Data Availability

No new data were created or analyzed in this study.
